# Infant versus noninfant formulas and cow's milk: Transition based on age or weight?

**DOI:** 10.1002/jpr3.12085

**Published:** 2024-06-21

**Authors:** Sarah Orkin, Kathryn Hitchcock, Jennifer Phillips, Emily Romantic, Amiee Trauth, Jacqueline Wessel, Marialena Mouzaki

**Affiliations:** ^1^ Division of Gastroenterology, Hepatology and Nutrition, Department of Pediatrics Cincinnati Children's Hospital Medical Center, University of Cincinnati College of Medicine Cincinnati Ohio USA; ^2^ Division of Nutrition Therapy Cincinnati Children's Hospital Medical Center Cincinnati Ohio USA

**Keywords:** electrolyte imbalance, feeding, iatrogenic, malnutrition

## Abstract

Infant formulas are meant to be used until 1 year of age, at which point children are transitioned to non‐infant formulas or cow's milk, depending on their remaining dietary intake. Noninfant formulas and cow's milk are appropriate for children who have an average weight at that 1‐year mark (9–9.5 kg); however, can contribute significant protein and/or electrolytes to children who are underweight for age, particularly if they still rely heavily on formula feeding for their caloric intake. In this short communication, we present several cases of patients who received excessive amounts of nutrients for age following the formula transition at the 1‐year mark. We also provide recommendations for clinicians to consider when faced with underweight infants who are meant to be transitioning off infant formulas.

## Abstract (highlights)

**What is Known:**

- Infant formulas are meant to be used within the first 12 months of life.
- At the 12‐month mark, it is recommended to transition off infant formulas, to either a noninfant formula or cow's milk.

**What is New:**

- Transition off infant formulas based on age alone can lead to significant electrolyte and acid‐based derangements in children who are significantly underweight for age.
- This may be due to excess nutrient delivery in the context of an immature gastrointestinal and/or renal physiology.
- Clinicians should consider transitioning children off infant formulas when both age (≥12 months) and weight (≥9–9.5 kg) criteria are met, particularly in patients with underlying medical conditions.

## 1 INTRDOUCTION

The American Academy of Pediatrics recommends offering human milk or infant formula until the age of 12 months with solids introduced at around 6 months of age.^1^ At the 1‐year mark, it is recommended that children transition to cow's milk, if they are consuming adequate amounts of solids (2–3 meals per day), or to non‐infant formulas, if they still rely heavily on formula to meet their calorie goals. According to the World Health Organization (WHO) growth charts, the average weight (50th percentile) of a 12‐month‐old child is 9–9.5 kg (females and males, respectively).^2^ However, a considerable number of children, particularly those with chronic medical issues, weigh significantly less than 9 kg at the 1‐year mark.^3^, ^4^ A significant deviation from the mean, as seen in cases of malnutrition, as well as underlying medical complexity, can have nutritional implications.

Most infant and noninfant formulas are designed to provide complete nutrition if offered in adequate amounts. Infant formulas, designed for those less than 1 year of age, have less protein (g/kilocalories (kcal)), potassium (mEq/kcal), and phosphorus (mmol/kcal) than noninfant formulas. This is because infants consume less solids and more formula (ml/kg/day) compared to their older counterparts.^5^ Conversely, noninfant formulas and cow's milk are higher in protein (g/kcal), as well as other electrolytes and minerals to provide optimal nutrition in smaller volumes. This is counterintuitive, as smaller children have increased caloric, macro‐, and micronutrient requirements, compared to their older counterparts (see details in Table 1).

**Table 1:** Average requirements of a 6 kg versus a 9 kg infant (RDA/AI).

	6 kg infant^a^	9 kg infant^b^
Protein (g/kg)	1.5	1.2
Sodium (mg/day)	120	370
Potassium (mg/day)	400	700
Calcium (mg/day)	200	260
Phosphorus (mg/day)	100	275

*Note*: Information retrieved from: https://www.canada.ca/content/dam/hc-sc/migration/hc-sc/fn-an/alt_formats/hpfb-dgpsa/pdf/nutrition/dri_tables-eng.pdf

Abbreviations: AI, adequate intake; RDA, recommended dietary allowance; WHO, World Health Organization.

^a^ Average weight for a 3‐month‐old per the WHO chart.

^b^ Average weight for a 12‐month‐old per the WHO chart.

When children rely on forms of nutrition that are outside the age norms (e.g., still relying solely on formula at 1 year of age) the standard, age‐based approach to transitioning from infant to noninfant formulas or cow's milk can lead to significant complications, particularly in those with underlying medical issues or immature physiology for age. For example, metabolic acidosis and/or renal injury can develop from excess protein provision, as well as potentially dangerous electrolyte abnormalities, such as hyperkalemia or hyperphosphatemia can be seen.^6^, ^7^, ^8^ Furthermore, nutritional deficiencies may develop if underweight children are transitioned to cow's milk at the recommended age cutoff (1 year of age). These concerns are heightened in subjects who have limited solid food intake and rely heavily on formula/milk for their nutrient delivery. The objective of this commentary is to underscore the limitations of the current approach of using the 1‐year of age cutoff to transition off infant formulas, using clinical examples. Furthermore, practical recommendations to address this issue are given.

## 2 CASE #1

A 12‐month‐old term female with a medical history notable for SOS2 mutation and ventriculoseptal defect was hospitalized for poor weight gain. At the time of admission, she weighed 6.16 kg (WHO weight z‐score −3.3) and was 64 cm long (WHO z‐score −4.1). Upon admission, she was receiving solely infant formula (Enfamil AR®) fortified to 24 kcal/ounce (oz) for a total of 756 kcal/day (945 mL/day). This formula provided 3.2 g/kg/day protein, 2.2 mEq/kg/day sodium, 3.4 mEq/kg/day potassium, and 65 mg/kg/day phosphorus. Due to her age and the desire to deliver more calories, she was switched to a noninfant formula (Kate Farms Pediatric Peptide 1.0®) with a new prescription that delivered 960 mL/day, 960 kcal/day, 5.8 g/kg/day protein, 4.5 mEq/kg/day sodium, 6.2 mEq/kg/day potassium, and 143 mg/kg/day phosphorus. Over the following 4 days, her serum potassium rose, from the admission baseline of 4.1 mmol/L up to 6.3 mmol/L (normal range for age: 3.3–4.7 mmol/L). Serum phosphorus increased from 5.1 to 6.3 mg/dL (normal range for age: 4.3–7.4 mg/dL). The serum bicarbonate levels dropped from 23 to 19 mmol/L during that time (normal range for age: 17–31 mmol/L). Due to persistent electrolyte abnormalities, she was switched back to an infant formula (Gerber HA®) 30 kcal/oz to deliver 850 kcal per day. This regimen provided 850 mL, 3.6 mEq/kg/day protein, 2.3 mEq/kg/day sodium, 3.4 mEq/kg/day potassium, and 86 mg/kg/day phosphorus. Over the following 3 days, the patient's serum potassium normalized, and phosphorus levels went back to baseline levels.

## 3 CASE #2

A 12‐month‐old term male with a history of type 1 laryngeal cleft s/p repair, dysphagia, silent aspiration, and poor weight gain was admitted to the hospital for nutritional rehabilitation. At the time of admission, he was 7.7 kg (WHO z‐score −2.1) and 71 cm (WHO z‐score −2.3). Before admission, the patient was on table foods plus reported intake of Enfamil Gentlease® 20 kcal/oz providing a total of 960 kcal/day, 2.9 g/kg/day protein, 2.2 mEq/kg/day sodium, and 3.5 mEq/kg/day potassium). Upon admission to the hospital, a nasogastric tube was placed and given his age, was switched to standard 30 kcal/oz Pediasure®. The new regimen provided similar calories at 950 kcal/day, as well as 3.6 g/kg/day protein, 2.0 mEq/kg/day sodium, and 6.2 mEq/kg/day potassium. Before the transition serum potassium was 4.6 mmol/L, and over the course of the next 3 days trended up steadily to 5.4 mmol/L. Due to persistent hyperkalemia, the enteral feed regimen was changed back to Enfamil Gentlease® but at a higher concentration than baseline (30 kcal/oz), providing 850 kcal/day, 2.5 g/kg/day protein, 1.9 mEq/kg/day sodium, and 3.1 mEq/kg/day potassium. With this change, the serum potassium trended down to 4.1 mmol/L.

## 4 CASE #3

This is a 12‐month‐old female born at 23 weeks gestation with a history of spontaneous intestinal perforation and chronic lung disease. Because this child had a prolonged course of parenteral nutrition and was considered to have mineral deficits, the plan was to keep her on a premature infant follow‐up formula (Neosure©) until 12 months corrected age. At 8 months corrected age her weight was 5.9 kg (WHO z‐score −2.52) and she was tolerating the preterm follow‐up formula at 24 kcal/oz. With that she was receiving 160 mL/kg, 755 kcal/day, 1.8 g/kg protein, 127 mg/kg calcium, and 80 mg/kg phosphorus. Serum phosphorus was 5.4 mg/dL (normal range for age: 3.8–6.5 mg/dL) and alkaline phosphatase was trending downward, now at 452 mg/dL (normal range for age: 122–469 U/L). At the 12‐month (chronological age, 8 months corrected age) Women, Infants, and Children (WIC) appointment her mother was given vouchers for cow's milk. On that, the intake at 160 mL/kg was 101 kcal/kg, 5.4 g/kg protein, 196 mg/kg calcium, and 161 mg/kg phosphorus. Due to feeding intolerance, caregivers called their provider who recommended going back to the previously tolerated formula. Thus, this patient remained on cow's milk for 3 days only. Laboratory studies were not obtained at that point. The medical team called the WIC office and the next month's vouchers were changed back to infant formula.

## 5 DISCUSSION

Here we provide several examples of the significant differences in nutrient delivery between infant and noninfant formulas (or cow's milk), as well as the potentially dangerous metabolic abnormalities seen when using noninfant formulas in children who are ≥12 months of age but weighing less than 9 kg and still rely heavily on formula feeding (limited solid food intake) due to underlying medical issues (Table 2). While these abnormalities are easily reversible with dietary modifications, a high index of suspicion is needed to test for these and to recognize that they are nutritional in nature.

**Table 2:** Changes in nutrient delivery when transitioning off infant formula.

	Formula	Energy (kcal/day)	Protein (g/kg/day)	Sodium (mEq/kg/day)	Potassium (mEq/kg/day)	Phosphorus (mg/kg/day)
Case #1	Infant formula	756	3.2	2.2	3.4	65
	Pediatric formula	960	5.8	4.5	6.2	143
Case #2	Infant formula	960	2.9	2.2	3.5	57
	Pediatric formula	950	3.6	2.0	6.2	65
Case #3	Infant formula	755	1.8	1.8	4.6	80
	Cow's Milk	596	5.4	3.4	6.0	161

As shown in Case #1, there are times when formula changes are made in conjunction with caloric increases. This is even more problematic, as a higher caloric delivery using a non‐infant formula leads to a disproportionately increased delivery of protein, potassium, and phosphorus. In Case #1, if the infant formula had been used to make the calorie increase desired (from 756 to 960 kcal/day), the potassium increase would have been more subtle (from 3.4 to 4.3 mEq/kg/day), as opposed to the 6.2 mEq/kg/day ultimately delivered with the non‐infant formula. Case #1 also highlights another important nutritional aspect, the fact that plant‐based formulas tend to have a higher protein content given the need to provide amino acids that are limiting in plant sources. This should be taken into consideration when choosing the optimal non‐infant formula to transition to.

Case #2 underscores the fact that even when aiming for similar calories, the transition to noninfant formulas in children with low weight‐for‐age can still cause electrolyte abnormalities. In this child, the abnormalities were noted promptly, and the formula was changed back to an infant product in 3–4 days. It is possible that even more significant abnormalities would have been seen, should the change have occurred in the outpatient setting where the monitoring is less frequent. Furthermore, neither of these cases represented patients with renal impairment, a population that is at even higher risk of electrolyte abnormalities in the context of increased electrolyte delivery.

Case #3 highlights that while government programs can sometimes dictate milk/formula choices, cow's milk is suboptimal for premature infants who have not yet reached 12 months of corrected age. This is particularly true in those who are also underweight for corrected age. The composition of the cow's milk may have contributed to the feeding intolerance seen in this context and is another reason to avoid transitioning to cow's milk until both the correct age and weight are reached.

## 6 SUMMARY AND RECOMMENDATIONS

Here we provide a series of examples of patients with underlying medical issues who were transitioned from infant to toddler formulas or cow's milk per the existing recommendation at 1 year of age and who developed preventable acid–base and electrolyte abnormalities, as they were too small for age. This report aims to address possible knowledge gaps in clinicians providing nutrition care to prevent such complications. Given the composition of infant versus non‐infant formulas or cow's milk, it is advisable to take weight into consideration, in addition to age, when transitioning children off infant formulas. This is particularly relevant for patients with chronic medical issues and children, who are thought to be physiologically immature for age. Specifically, considering that the average weight at 1 year of age is 9–9.5 kg, it is advisable to weigh the risks and benefits of transitioning an underweight child at 12 months of age and consider remaining on infant formula until that weight is reached. Furthermore, we recommend that the nutritional intakes of children with malnutrition are followed closely by trained dietitians, who can provide exact numbers with regard to macronutrient and micronutrient intakes. These, in turn, can inform decisions with regard to dietary prescriptions and the need for monitoring. This is particularly important for patients with chronic underlying medical issues whose physiology or treatments may prevent them from adapting to the excess nutrient delivery (e.g., via renal elimination). In the case of infants covered by WIC, providers should advocate for ongoing provision of noninfant formulas if the infants are not felt to be medically ready to transition. While fluid was not a focus of this commentary, fluid delivery changes depending on the type and calorie concentration of formulas and that should also be taken into consideration when deciding to transition off infant formulas.

## CONFLICT OF INTEREST STATEMENT

The authors declare no conflict of interest.
